# Rapid Detection of Benzo[a]pyrene in Extra Virgin Olive Oil Using Fluorescence Spectroscopy

**DOI:** 10.3390/molecules28114386

**Published:** 2023-05-27

**Authors:** Emmanouil Orfanakis, Aggeliki Koumentaki, Aikaterini Zoumi, Aggelos Philippidis, Peter C. Samartzis, Michalis Velegrakis

**Affiliations:** 1 Institute of Electronic Structure and Laser, Foundation for Research and Technology-Hellas (IESL-FORTH), 70013 Heraklion, Crete, Greece; morfanakis@iesl.forth.gr (E.O.); azoumi@iesl.forth.gr (A.Z.); filagg@iesl.forth.gr (A.P.); sama@iesl.forth.gr (P.C.S.); 2 Department of Materials Science and Technology, University of Crete, 70013 Heraklion, Crete, Greece

**Keywords:** olive oil, EVOO, benzo[a]pyrene, polycyclic aromatic hydrocarbons, PAH, optical spectroscopy, fluorescence spectroscopy

## Abstract

Extra virgin olive oil (EVOO) should be naturally free of polycyclic aromatic hydrocarbon (PAH) contamination. PAHs are carcinogenic and toxic, and may cause human health and safety problems. This work aims to detect benzo[a]pyrene residues in EVOO using an easily adaptive optical methodology. This approach, which is based on fluorescence spectroscopy, does not require any sample pretreatment or prior extraction of PAH content from the sample, and is reported for the first time herein. The detection of benzo[a]pyrene even at low concentrations in extra virgin olive oil samples demonstrates fluorescence spectroscopy’s capability to ensure food safety.

## 1. Introduction

Extra virgin olive oil (EVOO) has been declared a main component of the Mediterranean diet; it is correlated with health benefits due to its high nutritional value. These benefits can often be invalidated through contamination with pollutants. EVOO should be naturally free of contaminants such as polycyclic aromatic hydrocarbons (PAHs) because it is a fruit juice produced completely naturally from olives and without using chemical solvents or other additives. PAHs are a large group of hydrocarbon compounds composed of two or more aromatics rings. These compounds originate from incomplete combustion of organic matter that occurs from natural combustion, but are most likely due to man-made objects and activities, such as engine exhausts, industrial production and geological processes. Interest in studying PAHs is increasing, because exposure to their multiple potential sources of contamination and their carcinogenic and/or mutagenic properties can threaten humans health [1,2,3]. The contamination of EVOO by PAHs can occur directly, during the production line (in transportation, the mill, or mechanical processes) or indirectly from atmospheric deposition of PAHs onto the olive’s skin (through smoke or air pollution), which leads to contamination of the final product [4]. 

**Figure 1:**
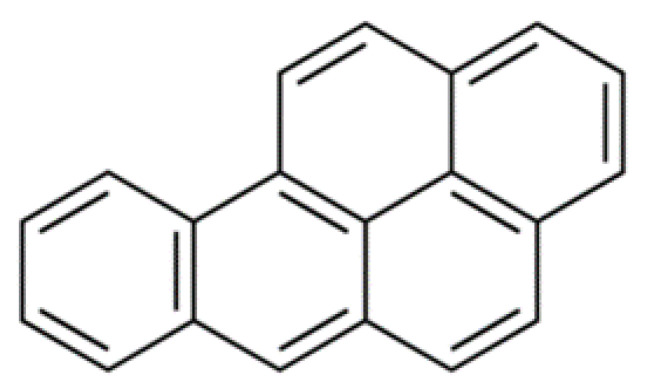
Chemical structure of benzo[a]pyrene.

Benzo[a]pyrene (BaP) (Figure 1) is the most known PAH due to its carcinogenic effects; until recently, it was the only PAH classified as a group 1 carcinogen by the International Agency for Research on Cancer [5]. In 2002, the Scientific Committee on Food of the European Commission recognized BaP as a suitable indicator of the presence and concentration of PAHs in foods [6]. Later, in 2006, the European Union in Regulation 1881/2006 defined the maximum limits for BaP in foods. The most recent regulation by the EU was released in 2011 (Regulation 835/2011), and it determines the maximum level of BaP to be 2 μg/kg and the maximum level of the sum of four PAHs (benzo[a]pyrene, benzo[a]anthracene, benzo[b]fluoranthene and chrysene) to be 10 μg/kg for oils and fats intended for direct human consumption, or for use as an ingredient in foods [7]. 

The low detection limit of PAH content and the diversity of potential interferences present in food is one of the principal problems associated with detecting PAHs. For this reason, preliminary extraction and cleanup procedures are required to isolate these compounds from EVOO and other edible oils. Techniques such as liquid–liquid extraction (LLE) with organic solvents, solid-phase microextraction (SPME), and microwave-assisted extractions are necessary to extract PAH from olive oil [4,8,9,10]. Analytical techniques have been used to determine the extracted PAH content, such as high-performance liquid chromatography (HPLC), with the use of several detectors, and gas chromatography coupled with mass spectrometry (GC-MS), which have been used for the detection of PAH content [4,8,10,11,12,13,14]. Specifically, liquid chromatography with fluorescence detection has been widely used to determine PAHs in a wide range of matrices [15,16,17,18,19].

Optical spectroscopy techniques provide a different perspective, and in recent years have frequently been used as an analytical approach with several applications in the agricultural sector [20,21,22,23,24]. Among the optical spectroscopic techniques, fluorescence spectroscopy is a potential tool for the determination of PAHs, due to their strong endogenous fluorescence. Moreover, this technique can also be portable, making it a versatile option for field studies [25]. This technique has been used for the detection of PAHs in water samples [26,27,28] and various products such as smoked tuna, fish oil, olive oil and other edible oils [29,30,31]. In the aforementioned studies, the fluorescence emission overlapping between the PAHs and the intrinsic fluorescent compounds contained in olive oil and other food products have led to the use of extraction or clean-up methods prior to fluorescence analysis. 

This study aims to determine benzo[a]pyrene content in EVOO samples using fluorescence spectroscopy. The advantages of this specific spectroscopic technique are that it is simple, rapid, and can detect benzo[a]pyrene at low concentrations without pretreatment and prior extraction.

## 2. Results and Discussion

**Figure 2:**
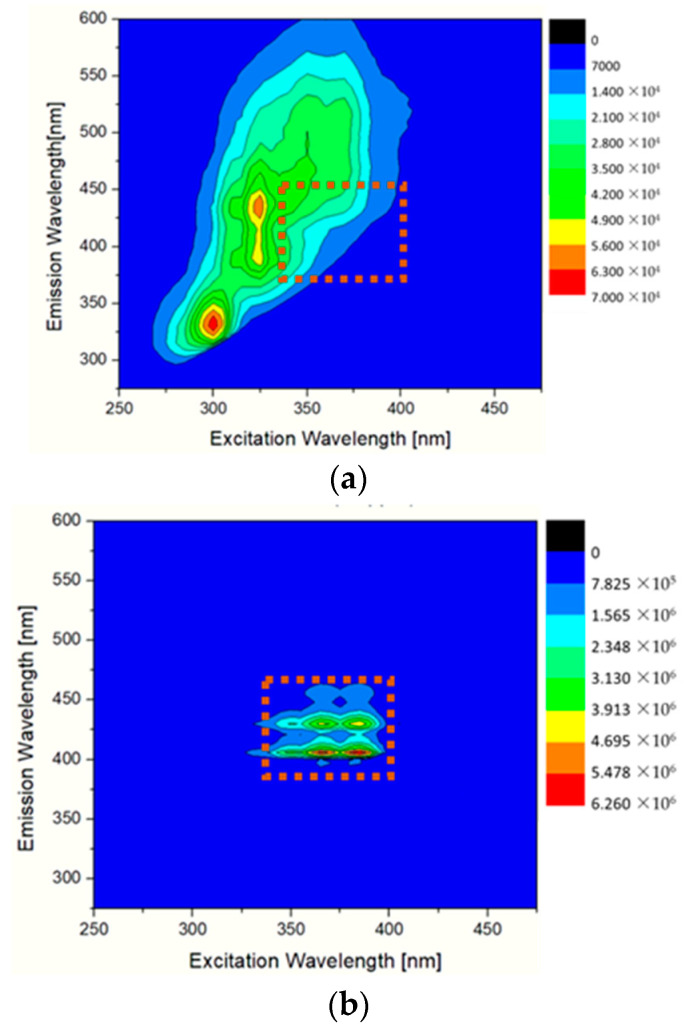
Fluorescence excitation–emission matrices (EEMS) of the (**a**) extra virgin olive oil and (**b**) benzo[a]pyrene stock solution in acetone (20 ppm).

EVOO and benzo[a]pyrene fluorescence excitation–emission matrices (EEM) were recorded by scanning both the excitation and emission monochromators. The excitation wavelength range for the matrices was set from 250 nm to 450 nm, and the emission wavelength range was set from 290 nm to 600 nm. Figure 2a is presented in the form of a contour plot of the EEM of a representative EVOO. The fluorescence regions that appear in Figure 2a are due to the several fluorophores contained in EVOO. The observed regions are associated with the tocopherols and their derivatives, phenolic compounds and oxidation products [32]. Since the range of emission wavelengths studied was between 290 nm and 600 nm, we do not detect the intense emission associated with the pigments, particularly chlorophyll, that have a maximum emission at approximately 670 nm [32,33]. The fluorescence region that appears in the BaP stock solution (Figure 2b) is located in the excitation wavelengths from 340 nm to 400 nm, and the emission wavelengths from 400 nm to 475 nm. Upon comparing the maps shown in Figure 2a,b, it is evident that there is minimal overlap between the fluorescence signals of EVOO and BaP. The fluorescence region corresponding to PAH is designated on both maps with dotted squares. For that reason, it is feasible to detect BaP in EVOO without any prior pretreatment or extraction.

**Figure 3:**
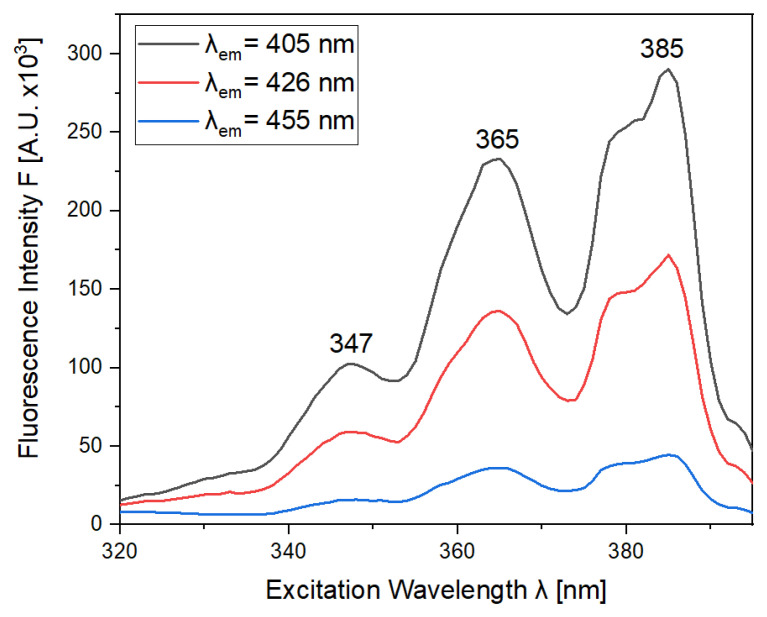
Fluorescence excitation spectra of benzo[a]pyrene in acetone (20 ppm) at emission wavelengths of 405 nm (black line), 426 nm (red line) and 455 nm (blue line).

From inspection of the excitation–emission matrices in Figure 2, it is evident that some excitation wavelengths are more appropriate to be selected for further exploration of the benzo[a]pyrene concentration in EVOO. This was accomplished with the investigation of the fluorescence excitation spectra that were recorded to determine the appropriate excitation wavelengths that produce the more intense emission signal for BaP, as shown in Figure 3. According to Figure 2b, the maximum fluorescence signal for BaP has been observed at wavelengths of 405 nm, 426 nm and 455 nm. At specific emission wavelengths, the corresponding excitation fluorescence spectra were recorded (Figure 3). As seen from the spectra, the excitation wavelengths at 347 nm, 365 nm and 385 nm are considered the most suitable for the fluorescence analysis of benzo[a]pyrene. However, the fluorescence of extra virgin olive oil overlaps with the PAH at the excitation wavelength of 347 nm, so this wavelength will be excluded from further analysis. 

**Figure 4:**
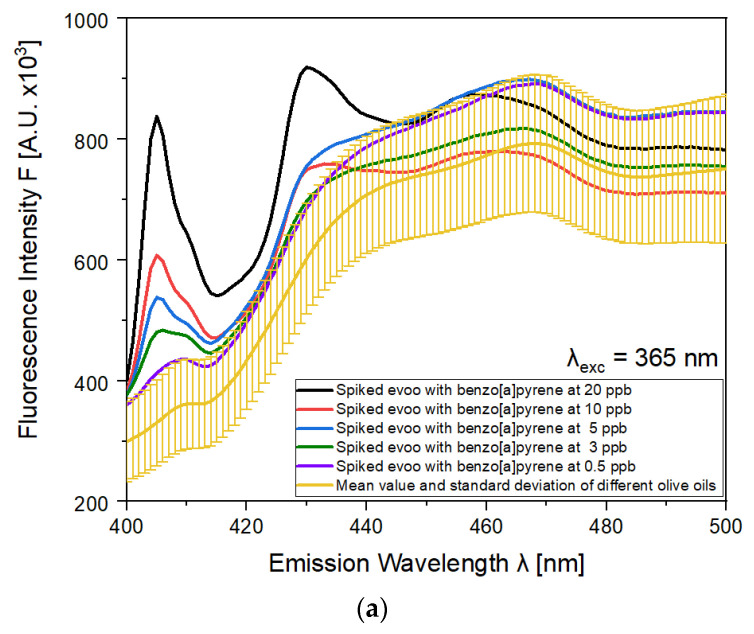

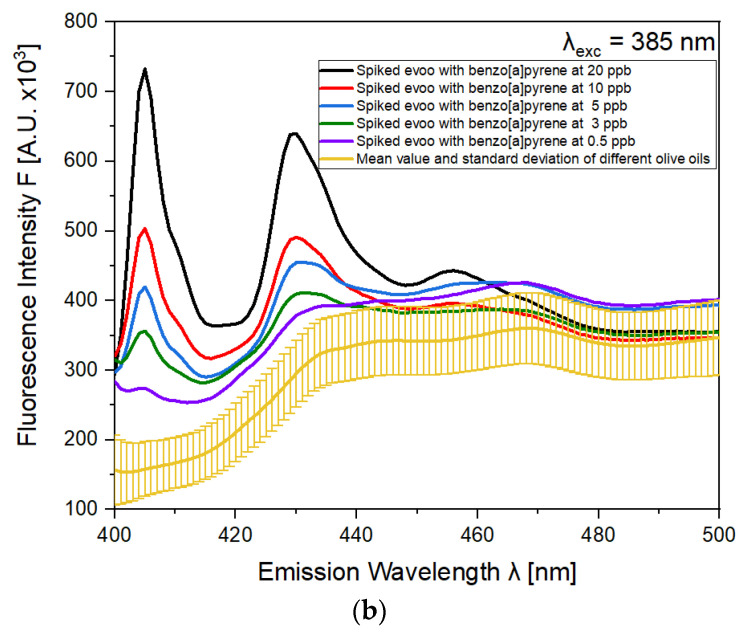
Fluorescence emission spectra of EVOO spiked with benzo[a]pyrene (concentration range 0.5–20 ppb) (**a**) at λ_exc_ = 365 nm (**b**) at λ_exc_ = 385 nm.

The fluorescence emission spectra of different BaP concentrations in EVOO at the selected excitation wavelengths are displayed in Figure 4. Specifically, Figure 4a shows the spectra with excitation at 365 nm, and the corresponding spectra at excitation wavelength 385 nm are presented in Figure 4b. In each figure, the yellow line depicts the average fluorescence spectra of 13 pure EVOOs, with their standard deviations for comparison. We observed that the mean value of the EVOO samples’ emission spectra have a standard deviation, which indicates differences in the offset of the samples. Moreover, as observed from the comparison of the different spectra, BaP presents intense emission bands from 400 to 450 nm. The intense fluorescence of the BaP solution that was observed is accomplished with both excitations (365 nm and 385 nm). The main advantage of the use of 365 nm and 385 nm excitation wavelength, is the absence of emission bands of olive oil in the range from 400 nm to 450 nm, in which the BaP appears to have an intense peak located at about 405 nm emission. In both spectra, this peak has higher fluorescence intensities compared with the peak of BaP located at 430 nm. 

**Figure 5:**
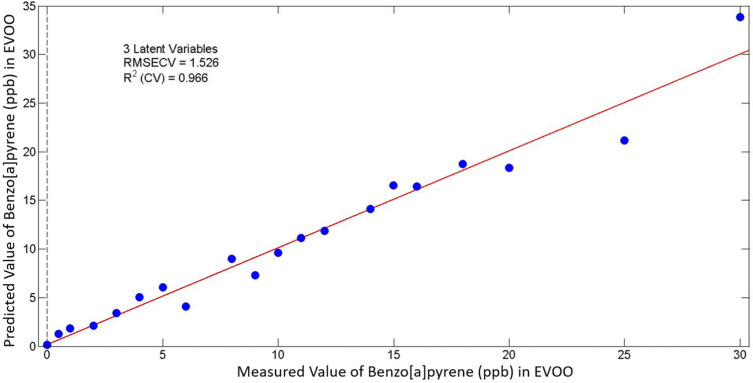
PLS regression model plot of predicted versus measured concentration of benzo[a]pyrene in EVOO based on fluorescence data for λ_exc_ = 365 nm and emission range 400–450 nm.

The fluorescence spectroscopic data have been processed with a partial least square (PLS) statistical model. The spectra have been preprocessed with first derivative and mean-centering. The first derivative is used to eliminate the EVOO samples’ high standard deviation. All the EVOO samples spiked with BaP were used to evaluate the model. The benzo[a]pyrene concentrations ranged from 0 ppb to 30 ppb. Additionally, to improve the performance of the PLS model and to reduce the irrelevant information for the emission of BaP, the range of the spectrum used was from 400 nm to 450 nm. The PLS statistical models that were employed were conducted from the different emission spectra that were obtained. As a result, two different PLS regression models were constructed, corresponding to the two different λ_exc_ that were used. Table 1 summarizes the statistical parameters of the PLS models (RMSECV and R^2^). As observed in Table 1, the performance of both constructed models results in high values of R^2^ for cross-validation. The best performance with higher values of R^2^ and lower values of RMSECV was accomplished with the use of λ_exc_ = 365 nm for the selected emission region from 400 nm to 450 nm. This PLS model presents a correlation coefficient (R^2^) of 0.966 and a root mean square error of cross-validation (RMSECV) of 1.526. Figure 5 displays the PLS regression model plot of the predicted versus measured concentration values of BaP in EVOO, based on the specific fluorescence spectroscopic data (for λ_exc_ = 365 nm). 

## 3. Materials and Methods

### 3.1. Samples, Chemicals and Reagents

Benzo[a]pyrene (≥96% purity) was obtained from Sigma-Aldrich, and acetone (≥99.8% purity) was purchased from Honeywell. The extra virgin olive oils (13 samples) that have been measured were obtained from the producers and were analyzed by Chemicotechniki Laboratory to determine the absence of PAH content. The benzo[a]pyrene stock solution was diluted in acetone at a concentration of 20 ppm. The spiked samples of BaP in EVOO were prepared at concentrations of 30, 25, 20, 18, 16, 15, 14, 12, 11, 10, 9, 8, 6, 5, 4, 3, 2, 1 and 0.5 ppb. All samples were stored at a low temperature (4 °C) in dark and brown glass vials until the analysis.

### 3.2. Instrumentation

A Jobin-Yvon Horiba Fluoro Max-P (SPEX) (HORIBA Ltd., Tokyo, Japan) spectrometer was used in this study to acquire fluorescence spectra. A quartz cuvette with a 10 mm path length was used for the measurements. To avoid inner filter effects and accomplish measurements without any pretreatment, the cuvette was placed at 35° in front-facing geometry to the incident beam. Moreover, the integration time was set at 1 s, and the excitation and emission bandwidths were 3 and 3 nm, respectively. All measurements were performed in triplicate. 

### 3.3. Statistical Analysis

The statistical method that was employed for the prediction of BaP concentration in EVOO was a partial least square (PLS) regression analysis. PLS is a supervised method that is widely used to build statistical models with relations between measured and predicted values. For the PLS models that were constructed, all samples in the data sets were used as the training set. The validation of the models was performed using cross-validation (Venetian blinds), and the results that are presented herein are from after cross-validation.

The results are presented in the form of prediction plots. The predictive ability of the model is evaluated by the correlation coefficient (R^2^) and the root mean square error of cross-validation (RMSECV). The preprocessing technique that was used for the fluorescence spectroscopic data was the derivative and mean center. 

Data analysis was carried out using Matlab R2013b (Mathworks, MA, USA) with the PLS Toolbox 8.1 (Eigenvector Research, Manson, WA, USA).

## 4. Conclusions

In this work, benzo[a]pyrene in different concentrations (ppb) in EVOO has been detected using fluorescence spectroscopy. The application of fluorescence spectroscopy was performed without any sample extraction or pretreatment, and to the best of our knowledge, for the first time in the literature. The spectroscopic results were evaluated using high-performance PLS regression models. Fluorescence spectroscopy enables the detection of benzo[a]pyrene in EVOO samples at low concentrations, ensuring its concentration is consistent with EU regulations, which stipulate a maximum level of 2 ppb for olive oils.

The findings of the present study provide a useful alternative tool to directly detect benzo[a]pyrene residuals in EVOO samples, with a rapid, cost-effective technique that does not require any extraction of the target analyte in EVOO. Further studies may include the expansion of the methodology for the detection of a wide range of PAHs in EVOO, and the adaption of the present technique for on-site application.

## Figures and Tables

**Table 1:** Statistical parameters (RMSECV, R^2^) of the constructed PLS regression model for the prediction of the concentration of benzo[a]pyrene in EVOO based on fluorescence data.

Model		Statistical Parameters	
λ_exc_ (nm)	Selected Region (nm)	R^2^	RMSECV
365	400–450	0.966	1.526
385	400–450	0.957	1.753

## Data Availability

Not applicable.
